# Innovation in Aircraft Cabin Interior Panels. Part II: Technical Assessment on Replacing Glass Fiber with Thermoplastic Polymers and Panels Fabricated Using Vacuum Forming Process

**DOI:** 10.3390/polym13193258

**Published:** 2021-09-24

**Authors:** Edgar Adrián Franco-Urquiza, Perla Itzel Alcántara Llanas, Victoria Rentería-Rodríguez, Raúl Samir Saleme, Rodrigo Ramírez Aguilar, Cecilia Zarate Pérez, Mauricio Torres-Arellano, Saúl Piedra

**Affiliations:** 1 National Council for Science and Technology, Center for Engineering and Industrial Development (CONACYT–CIDESI), Carretera Estatal 200, km 23, Querétaro 76265, Mexico; saul.piedra@cidesi.edu.mx; 2 Center for Engineering and Industrial Development (CIDESI), Carretera Estatal 200, km 23, Querétaro 76265, Mexico; perla.alcantara@cidesi.edu.mx (P.I.A.L.); ana.renteria@cidesi.edu.mx (V.R.-R.); raul.saleme@cidesi.edu.mx (R.S.S.); rodrigo.ramirez@cidesi.edu.mx (R.R.A.); cecilia.zarate@cidesi.edu.mx (C.Z.P.); mauricio.torres@cidesi.edu.mx (M.T.-A.)

**Keywords:** aircraft cabin interior panels, vacuum forming, non-structural composite panels, thermoplastic face sheets

## Abstract

The manufacturing process of the aircraft cabin interior panels is expensive and time-consuming, and the resulting panel requires rework due to damages that occurred during their fabrication. The aircraft interior panels must meet structural requirements; hence sandwich composites of a honeycomb core covered with two layers of pre-impregnated fiberglass skin are used. Flat sandwich composites are transformed into panels with complex shapes or geometries using the compression molding process, leading to advanced manufacturing challenges. Some aircraft interior panels are required for non-structural applications; hence sandwich composites can be substituted by cheaper alternative materials and transformed using disruptive manufacturing techniques. This paper evaluates the feasibility of replacing the honeycomb and fiberglass skin layers core with rigid polyurethane foams and thermoplastic polymers. The results show that the structural composites have higher mechanical performances than the proposed sandwich composites, but they are compatible with non-structural applications. Sandwich composite fabrication using the vacuum forming process is feasible for developing non-structural panels. This manufacturing technique is fast, easy, economical, and ecological as it uses recyclable materials. The vacuum forming also covers the entire panel, thus eliminating tapestries, paints, or finishes to the aircraft interior panels. The conclusion of the article describes the focus of future research.

## 1. Introduction

The interiors of aircraft cabins play an essential role in improving the comfort of passengers on board [1,2,3]. Based on the type of interior, the aircraft cabin interior market has been segmented into aircraft seating, inflight entertainment and connectivity, cabin lighting, galley equipment, aircraft lavatories, aircraft windows and windshields, overhead compartments, and aircraft interior panels [4,5]. The current cabin interior industry is moving to modernize cabin interiors satisfying three main criteria: commercial viability, certification, and passenger experience. Some of the most notable moves are the development of lightweight and custom cabin solutions to enhance the overall passenger experience; an expansion of the overhead storage compartments to accommodate more luggage per passenger; and the design of a maximum cabin space that allows comfort and the feeling of space, while optimizing the space to add more seats. In this sense, composite materials represent an indispensable focus area for the aircraft cabin design without sacrificing performance, safety, and cost.

Aircraft seats are the most demanded product in the aircraft cabin interior market, while galley equipment is the most expensive. The interior panels of aircraft cabins are a combination of different products, and each product has its function. The aircraft interior panels are floor panels, roof panels, side panels, and cabin dividers [1,6,7,8]. The requirements of the aircraft cabin interior panels present a good balance between strength, low density, high stiffness, and durability [9,10,11,12,13]. In addition, most panels require contoured forms, which lead to several defects induced during the manufacturing process. Aircraft cabin interior panels are manufactured using typical sandwich composites configuration made with aramid honeycomb core bonded to outer layers or face sheets, commonly glass fiber epoxy prepreg. Lightweight, low-density core between thin sheets dramatically increases panel rigidity with little added weight. Complex panels are fabricated using both single-opening and multi-opening presses. For molds with complex geometries, the one-step compression molding leads to distinct types of induced failures, such as breaks, holes, and the absence of impregnated zones or areas with excessive resin accumulation in the contours of the mold [14]. The previous occurs when the sandwich composite is placed in a large press and crushed to a predetermined shape and thickness [15]. Then, the crushed honeycomb acts as multiple blades that break the glass fiber prepreg during the transformation process. Several research articles deal with the evaluation of sandwich composite fabrication [13,15,16,17,18]. However, the replacement of fiberglass layers with thermoplastic polymers and the substitution of honeycomb for structural foams oriented to the aircraft cabin interior panels are not profoundly studied, and this is the gap this work pretends to fill.

A previous work entitled “Innovation in aircraft cabin interior panels part I: Technical assessment on replacing the honeycomb with structural foams and evaluation of optimal curing of prepreg fiberglass” [19] was focused on the evaluation of the primary components for structural sandwich panels such as aramid paper honeycomb, vinyl foam, and glass fiber prepreg skin. In addition, the paper evaluates the optimal curing processing parameters through the Differential Scanning Calorimetry (DSC) technique by varying the processing manufacturing conditions.

This work evaluates the feasibility of using thermoplastic polymers to replace the fiberglass faces in the foam core to produce alternative aircraft interior panels.

## 2. Materials

The sandwich panel used as reference consists of honeycomb core faced with two skin plies of fiberglass fabric epoxy prepreg DA 4080 purchased to Adhesive Prepregs for Composite Manufacturers (APCM, Plainfield, CT, USA). DA 4080 is a one-sided pre-impregnated E-glass made up of a 200 gsm layer of bidirectional fabric and 3702 filaments/yarn. DA 4080 is a 200 °F low-temperature cure recommended for laminating and honeycomb sandwich construction. In this work, the glass fiber was labeled as GF.

The core used was the PN1 honeycomb, manufactured by DuPont Nomex^®^ (Wilmington, DE, USA) and purchased to Plascore. The PN1 is a sheet with regular hexagonal cell configuration, approximately 0.5-inch/13 mm thick, and a 0.12-inch/3 mm cell size.

In this work, the PN1 honeycomb (HC) is replaced by the 3 lb. density lightweight DIAB 1022 Vynil foam Divinycell^®^ (Helsingborg, Sweden) supplied by Fibreglast. According to the supplier, the DIAB 1022 (Foam) offers the highest strength to density ratio, insulative properties, improved stiffness, and impact resistance. The 0.5 (12.7 mm) thick foam is best when only additional impact resistance is required. This foam can be easily thermoformed with a heat gun or oven. Other properties of this foam are excellent adhesion/peel strength, chemical resistance, good dimensional stability, and temperature performance. The operating temperature can reach from −200 °C to 70 °C, although the processing temperature depends on time and pressure conditions.

The Acrylonitrile–Butadiene–Styrene (ABS) is a plastic used in engineering due to its excellent combination of mechanical properties. ABS is a two-phase terpolymer; one is a styrene–acrylonitrile rigid copolymer, and the other is a styrene–butadiene rubber-like copolymer. The plastic name is derived from the three initial monomers, which are mixed in different proportions.

Clariant Renol Gray ABS with 30% fiberglass of weight content as reinforcement was used in this work. According to the supplier, the ABS has molding temperatures between 65.6–93.3 °C, a specific gravity of 1.04 cm^3^, a tensile stress of 39.3 MPa, elongation of 40%, and a flexural modulus of 20.7 GPa.

**Figure 1:**
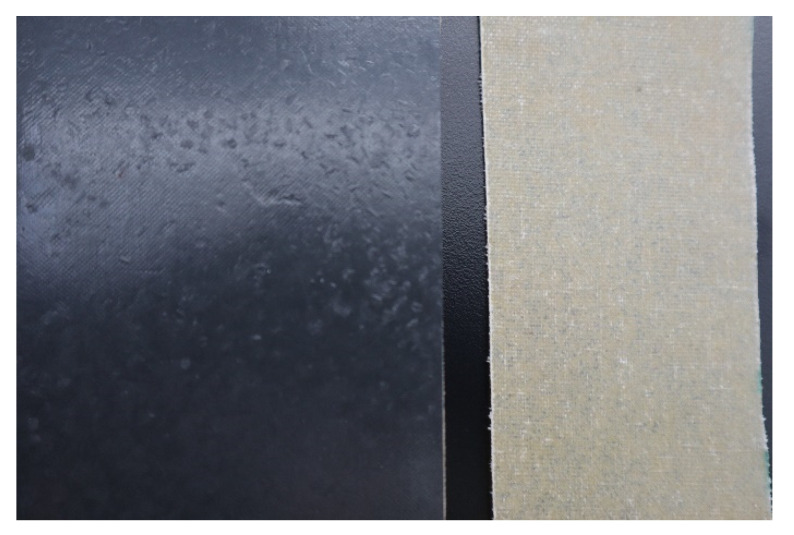
Pictures of the ABS (**left**) and GF prepreg (**right**) sheets used to manufacture distinct sandwich composite panels.

Figure 1 shows the GF prepreg and the ABS sheets before using to form the panels. The honeycomb and foam cores were presented in a previous work [19].

This work will manufacture prototype panels using a V-shaped mold to simulate one of the complex shapes manufactured in the aeronautical cabin interiors sector. The mold was made of aluminum 6061-T6 and adapted to a CARVER model 4122 hot plate press to fabricate composite sandwich panels at 256 °F with a constant pressure of 20 psi for one hour.

Composite materials are characterized by being designed according to their final application and the physical, mechanical, and chemical phenomena to which they will be exposed. The configuration of panels or sandwich composites stands out in structural applications thanks to their properties against compression and bending forces [20,21]. These panel structures were evaluated following the procedure indicated in the ASTM C393/C393M–20 [22], which considers the stresses that affect the mean span of 150 mm length in a flat specimen with suggested dimensions of 75 mm in width and 203 mm in length. ASTM C393 indicates that the results must be reported through the load versus crosshead displacement plots to determine if there is any significant compliance change (change in slope of the force–displacement curve, sometimes referred to as a transition region) before ultimate failure. However, for non-standard configurations, it is suggested that the width should not be less than twice the total thickness nor more than six times the total thickness [23,24]. The bending tests were performed in a universal testing machine MTS Insight with a cell load of 30 kN at room temperature and a crosshead speed of 0.5 mm/min.

**Figure 2:**
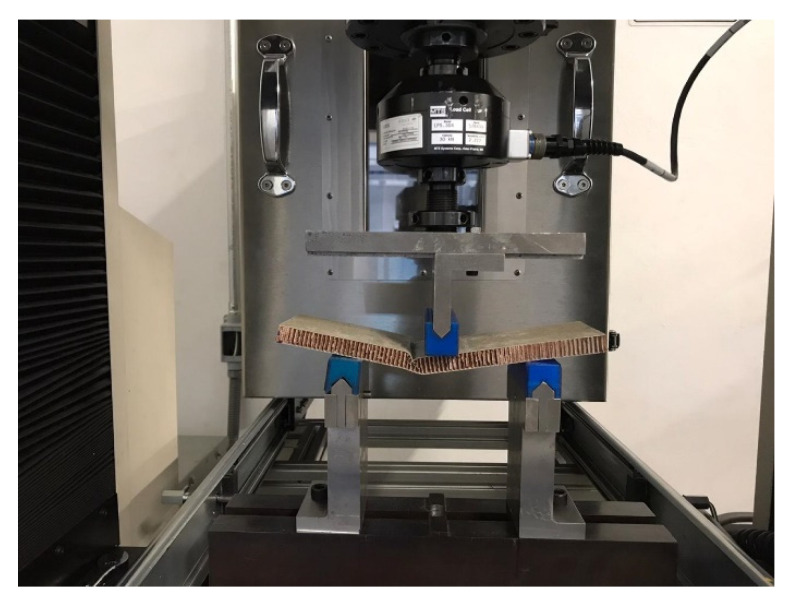
Photograph corresponding to the arrangement for the flexural test in reference panels according to the ASTM C393/C393M-20 standard.

Two support bars are placed under the sample, and a force is applied vertically on the specimen (Figure 2).

The distance between the support bars should be 150 mm (6.9 in). Pressure pads are used between the load bars and the specimen to prevent localized damage to the faces of the specimen. The following equation is used to calculate the maximum shear stress of the core:

(1) $$F_{s}^{ult}=\frac{P_{max}}{\left(d+c\right)\cdot b}$$

where $F_{s}^{ult}$ represents the maximum shear stress of the core in MPa, *P_max_* is the maximum force prior to failure (N), *d* is the thickness of the sandwich (mm), *c* is the thickness of the core (mm, *c* = *d* − 2*t*), *t* the nominal thickness of the faces (mm), and *b* the width of the sandwich (mm).

For core materials that have a yield above 2% strain, the yield shear stress equation should be used:

(2) $$F_{s}^{yield}=\frac{P_{yield}}{\left(d+c\right)\cdot b}$$

where $F_{s}^{yield}$ is the maximum core shear stress (MPa), and the $P_{yield}$ is the force at 2% offset shear strain (N). To calculate the stress of the faces:

(3) $$\sigma =\frac{P_{max}\cdot S}{2t\cdot \left(d+c\right)\cdot b}$$

where *t* is the thickness of the faces (mm) and *S* the length of the span (mm).

The sandwich composite panels evaluated in this work are listed in Table 1.

## 3. Manufacturing Panels

**Figure 3:**
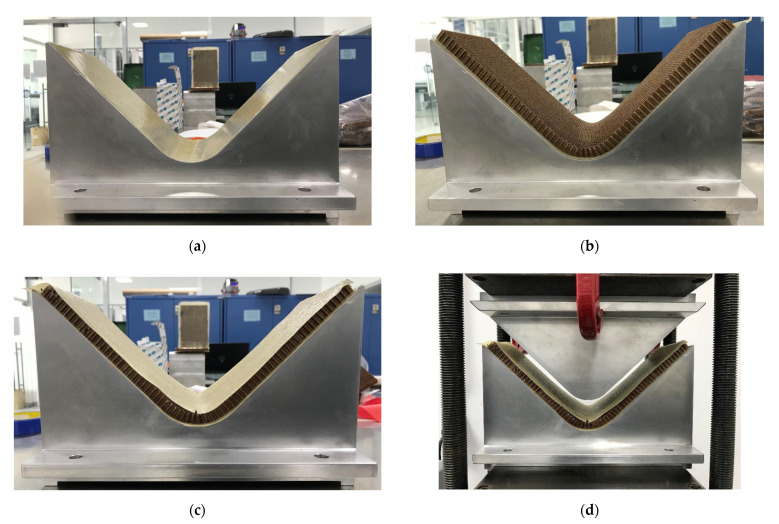

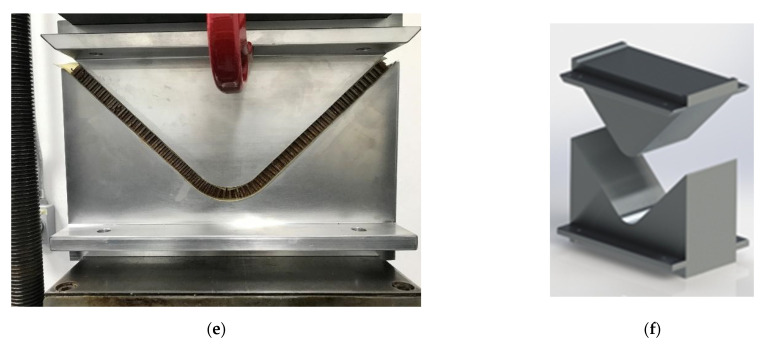
Schematic representation of the V-shaped mold to produce composite sandwich panels: (**a**) Two layers of GF prepreg (step two), (**b**) HC placement (step three), (**c**) complete sandwich structure (step three), (**d**) preheated process (step four), (**e**) manufacturing of V-shaped panel (step five), and (**f**) digital rendering of the V-shaped mold.

The manufacturing of V-shaped composite panels consisted of the following steps:

1. Three layers of mold release agent were applied to the two components of the V-shaped mold. With that covered, two layers of prepreg fiberglass were carefully stacked on both faces of the V-shaped mold, as presented in Figure 3.
2. Two layers of GF prepreg were placed on one of the faces of the V-shaped mold.
3. The HC specimen was placed on the GF prepreg layers, and two additional layers were placed on the top of the HC to build the sandwich composite configuration.
4. The mold is closed slowly to transfer the temperature from the hot plates to the V-shaped mold. The preheated process consisted of 256 °F during 30 min.
5. After the preheated process, the mold is closed, supplying a constant pressure of 20 psi and a temperature of 256 °F for one hour. Digital temperature recorder and thermocouples on both sides of the V-shape mold were used to monitor and verify the adequate pressure and temperature during the curing process.

The manufacturing process was applied to all configurations listed in Table 1.

**Figure 4:**
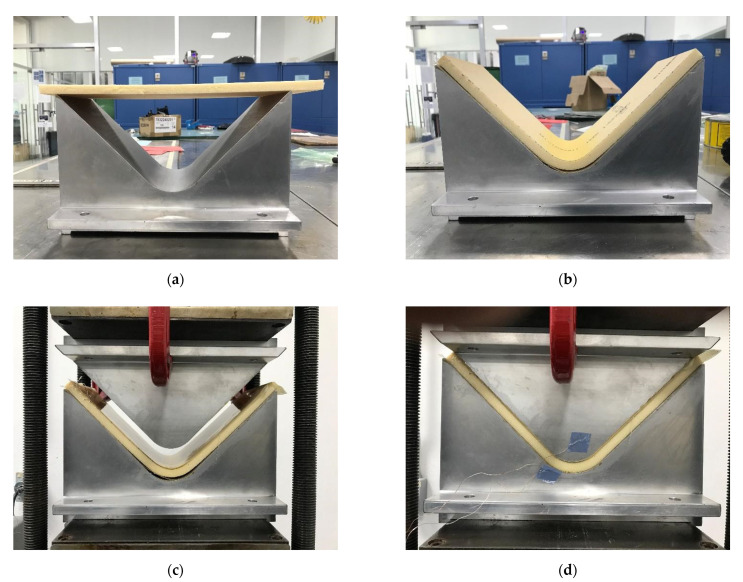
Schematic representation of the V-shaped mold to produce the foam sandwich panels: (**a**) Unbending foam specimen, (**b**) foam specimen adapted to the V-shaped mold, (**c**) preheated process, and (**d**) manufacturing of V-shaped foam panel.

It is essential to underline that the HC is flexible and easy to place and adapt to the V-shaped mold. However, the foam is a rigid structure, and it is impossible to blend at room temperature without damage or breaking the specimen. Therefore, two strategies were followed. The first one uses a heat gun to apply heat to the foam surface to soften the material. After that, the foam specimen can be mold into the V-shaped mold, as shown in Figure 4.

**Figure 5:**
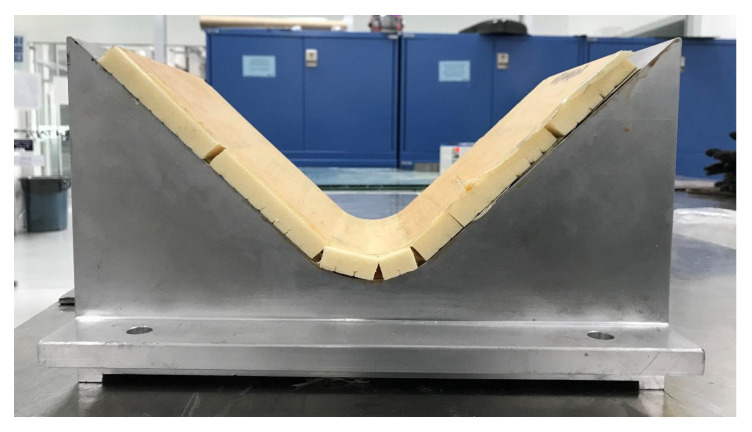
Picture corresponding to the gridding foam specimen placement in the V-shaped mold before the preheated process.

The second strategy consisted of cutting the foam specimen in a grid, without completing the cut, just enough to bend the foam specimen in different directions, as presented in Figure 5.

**Figure 6:**
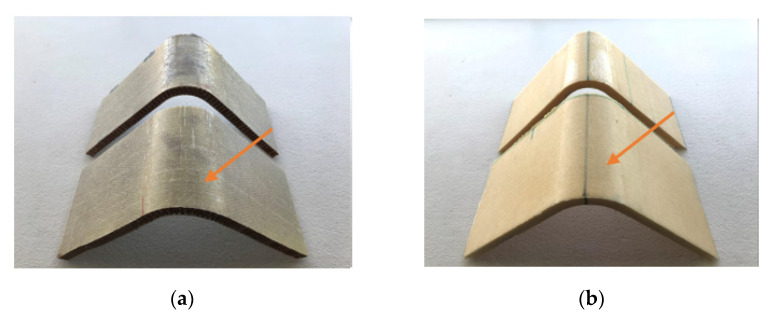

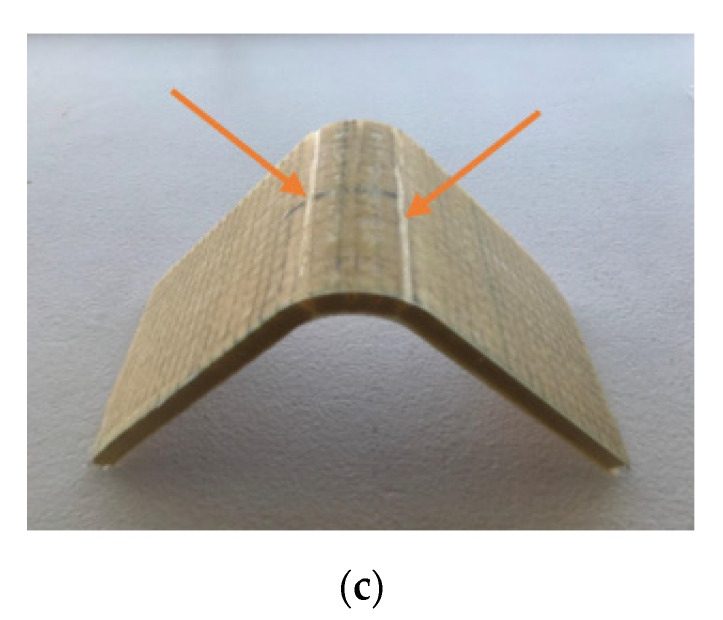
Pictures corresponding to the V-shaped panels of: (**a**) GF/HC/GF, (**b**) GF/Foam/GF, and (**c**) GF/griding foam/GF.

The gridding foam allows its correct arrangement on the walls of the mold, avoids the material having to be softened with heat to bend it, and its handling when placing the pre-impregnated fiberglass saves time. However, from Figure 5, it can be seen that the gridding foam does not cover the entire surface of the mold, leaving spaces that are not in solid contact with the fiberglass that could weaken the structure of the panel [25]. Figure 6 shows the sandwich panels prepared.

It is possible to appreciate the base of the V-shaped panels presenting similar features such as the build-up of epoxy resin in the top fold, indicated by an arrow in Figure 6a,b. This defect is attributed to the non-homogeneous contact between core and GF prepreg during the panel fabrication. The core is deformed to fit the V-shaped mold. This defect is not observed in the panel made with gridding foam as it has more significant contact with the fiberglass. However, the gridding foam panel shows an evident defect, indicated by arrows in Figure 6c, which occurs due to the non-contact of the GF prepreg, as shown more evident in Figure 5.

## 4. Results

**Figure 7:**
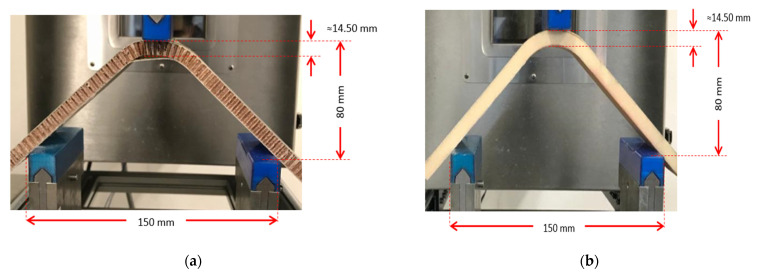

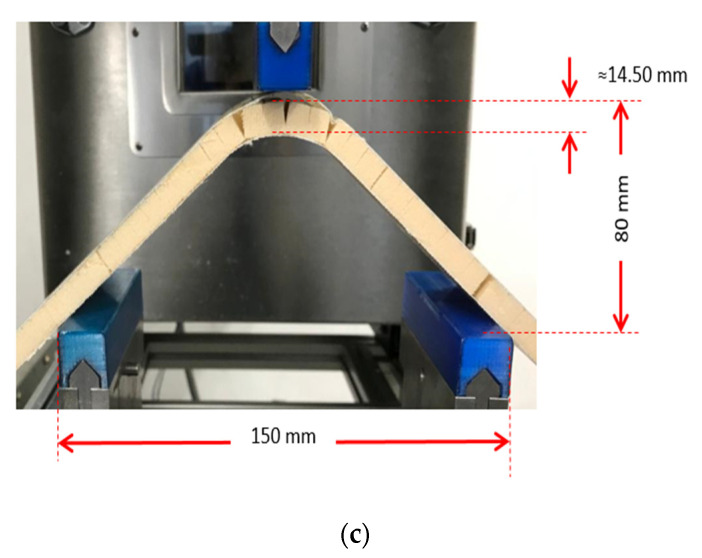
Pictures of the three-point bending test performed to: (**a**) GF/HC/GF, (**b**) GF/Foam/GF, (**c**) GF/Gridding foam/GF. The pictures present the damages that occurred during the test.

The GF/HC/GF, GF/Foam/GF, and GF/Gridding foam/GF systems were evaluated according to the ASTM C393. The panels were placed in a three-point bending configuration, with a span of 150 mm and a crosshead speed of 1 mm/min. The mechanical behavior of the panels is shown in Figure 7.

During the bending test, the GF/HC/GF reference composites showed high resistance to breakage, remaining firm until the fiberglass skin debonds. Similarly, the experimental composites GF/Foam/GF showed resistance to delamination. However, a failure of the foam core was noted, showing cracks and subsequent failure of the debonding between the fiberglass skin and the core.

As might be expected, the experimental GF/Gridding foam/GF laminate presented a collapse of the structure, attributed to the empty areas produced by the gridding (as noted in Figure 5), which weakened the structure [26].

**Figure 8:**
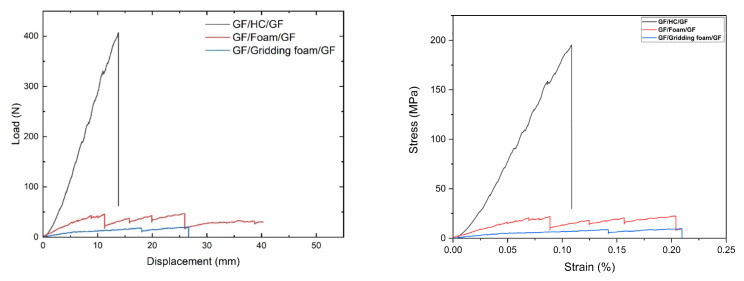
Representative load vs. displacement (**left**) and stress vs. strain (**right**) curves registered during the bending tests of GF/HC/GF, GF/Foam/GF, and GF/Gridding foam/GF panels.

Figure 8 shows the load versus displacement curves obtained from the bending tests for the GF/HC/GF, GF/Foam/GF, and GF/Gridding foam/GF systems. Table 2 presents the mechanical parameters determined. The ASTM C393/C393M–20 requires the load versus displacement plots from representative specimen behavior. The stress versus strain curves, calculated following the procedure proposed by Barbero et al. [27] are also added.

**Figure 9:**
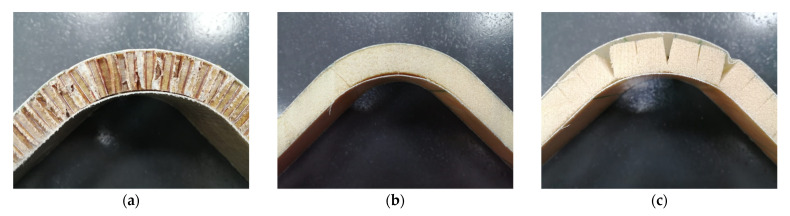
Pictures corresponding to the V-shaped panels after bending tests of (**a**) GF/HC/GF, (**b**) GF/Foam/GF, and (**c**) GF/griding foam/GF.

It is evident that better mechanical performance is obtained with the GF/HC/GF panel (Figure 9a), which is attributed to the intrinsic mechanical properties of the honeycomb structure and the molecular compatibility between the aramid in the HC and the epoxy resin from the GF prepreg [17]. The foam is a rigid material with limited mechanical performance compared to the HC. According to Figure 9b, the primary damage of the GF/Foam/GF panel is the failure of the foam and not the adhesive, which could be associated with some thermal degradation induced with the heat gun previous during the manufacturing process [17]. The GF/Gridding foam/GF panel is interesting as this material has the lowest bending properties, as expected, but avoids possible foam degradation that influences the shear strength properties (Table 2). This panel fails with the adhesive delamination, similar to the GF/HC/GF panel, as observed in Figure 9c.

The ABS sheets were molded using a square frame of 300 × 300 × 1 mm thick, previously calculating the volume of the square frame considering the density of the ABS (1.03 g/cm^3^) according to the technical datasheet. The ABS sheets were prepared in the CARVER hot plate press model 4122 at a temperature of 200 °C and pressure of 20 psi for 3 min. The unloading and loading procedure was performed to eliminate trapped air. Subsequently, the square frame was placed in a plate press at 30 °C and pressure of 200 psi for 2 min to cool the ABS sheets.

Several methodologies were followed to prepare ABS/foam/ABS and ABS/HC/ABS flat panels in just one step. However, it was not possible to adhere the ABS to the foam or to the HC. The methodologies are not the scope of this work.

**Figure 10:**
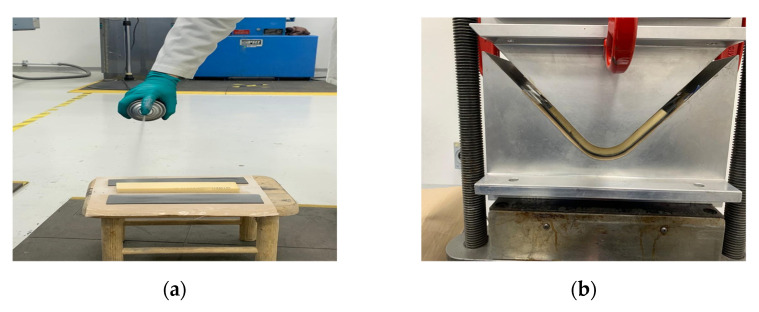

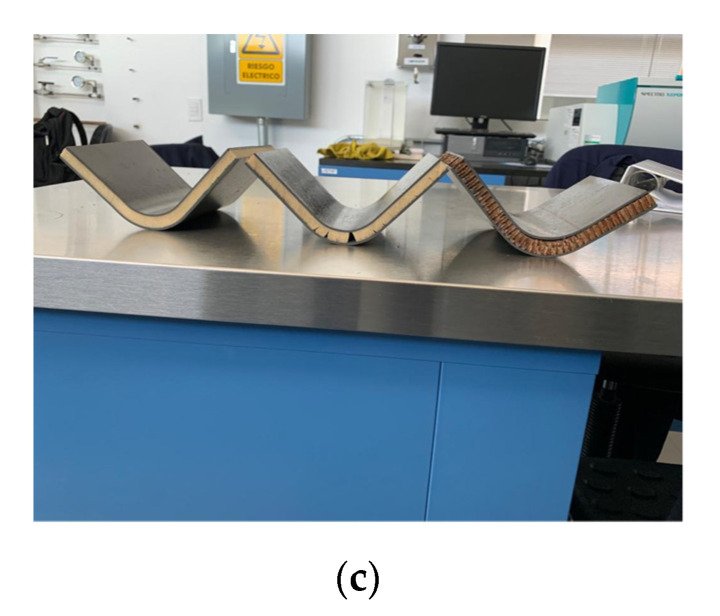
Pictures corresponding to the manufacturing procedure of V-shaped ABS panels: (**a**) spraying adhesive mist aerosol to the foam and ABS sheets, (**b**) ABS/foam/ABS compression molding process, and (**c**) V-shaped sandwich panels with face ABS sheets.

Therefore, the foam and HC specimens were sprayed with Hi-Tack 71 from 3M™, a mist aerosol adhesive recommended by 3M for its use for the manufacturing composites, including infusion and dry lamination. Spraying was carried out at a 45° angle before ABS sheets placement and subsequently put in the V-shaped mold to proceed with the panel manufacturing, as shown in Figure 10.

**Figure 11:**
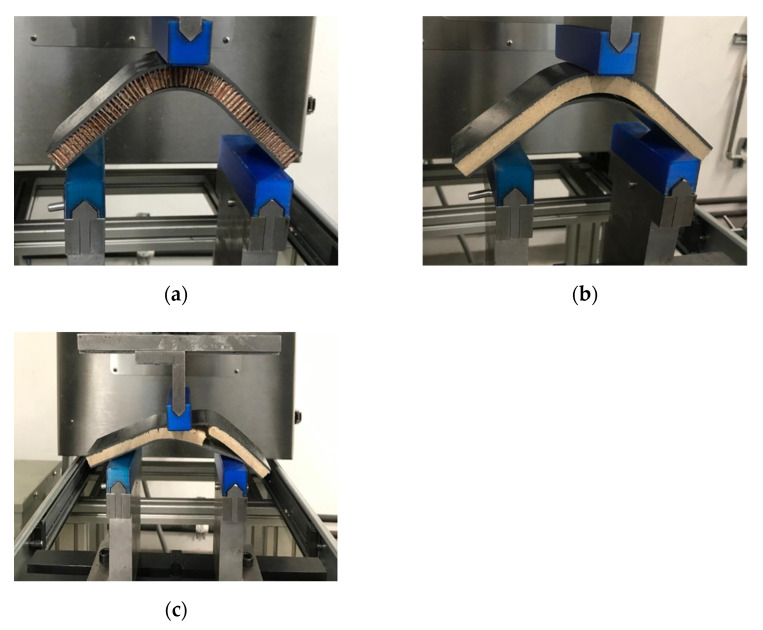
Pictures of the three-point bending test performed to (**a**) ABS/HC/ABS, (**b**) ABS/Foam/ABS, and (**c**) ABS/griding foam/ABS. The pictures present the damages that occurred during the test.

Figure 11 shows the bending tests of the sandwich composites using ABS as face sheets. Due to the restrictions in the size of the compressed molded ABS sheets, the span was fit to 120 mm. The mechanical parameters are reported in Table 2.

Based on the results, it is possible to suggest that the core acts actively more than the panels with GF prepreg in panels with ABS as face sheets. After the first delamination of the ABS, the load applied is supported by the core. That is the reason the ABS/HC/ABS presents lower mechanical performance than ABS/Foam/ABS. The foam is more rigid than HC. Considering the V-shape of the specimens, the HC under this configuration is less restrictive than the foam.

**Figure 12:**
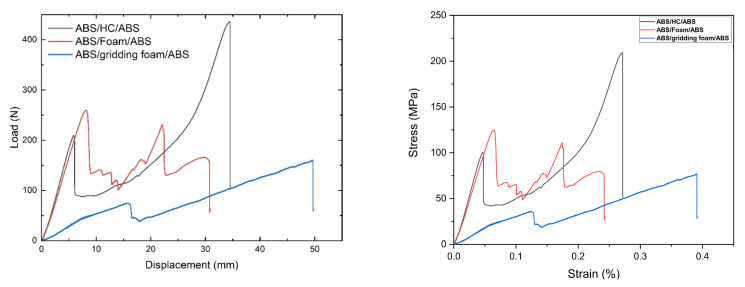
Representative load vs. displacement (**left**) and stress vs. strain (**right**) curves registered during the bending tests of: ABS/HC/ABS (green line), ABS/Foam/ABS (red line), and ABS/gridding foam/ABS panels (blue line).

Nonetheless, the spry adhesive seems to be more effective in the foam than HC, as the ABS/Foam/ABS supports higher load values (Figure 12). The previous is attributed to the surface contact, higher in the foam than HC structure. Similar is observed with the gridding foam, which loses rigidity and surface contact during cutting—the more flexibility, the lower mechanical resistance.

Thermoforming, or vacuum forming, is a process where a flat thermoplastic sheet is heated and molded into the desired shape. The process is widely used for packaging consumer products and making large items such as bathtubs, contour reflectors, and interior door liners for refrigerators.

It is considered as a secondary forming process; the primary process is when molded sheets or films. Only thermoplastics can be thermoformed, as extruded sheets of thermoset or elastomeric polymers are already cross-linked and cannot be molded by reheating.

In this work, the vacuum forming process was used to build panels oriented to the aircraft interiors. The panels were fabricated in a vacuum-formed machine Formech 508FS (Floor-Standing) with reduced window process, and quartz heaters for better forming performance. The optimal conditioning parameters for vacuum formed were 6 bar of vacuum pressure, 50% of power quartz heaters, and 160 s of heating time.

**Figure 13:**
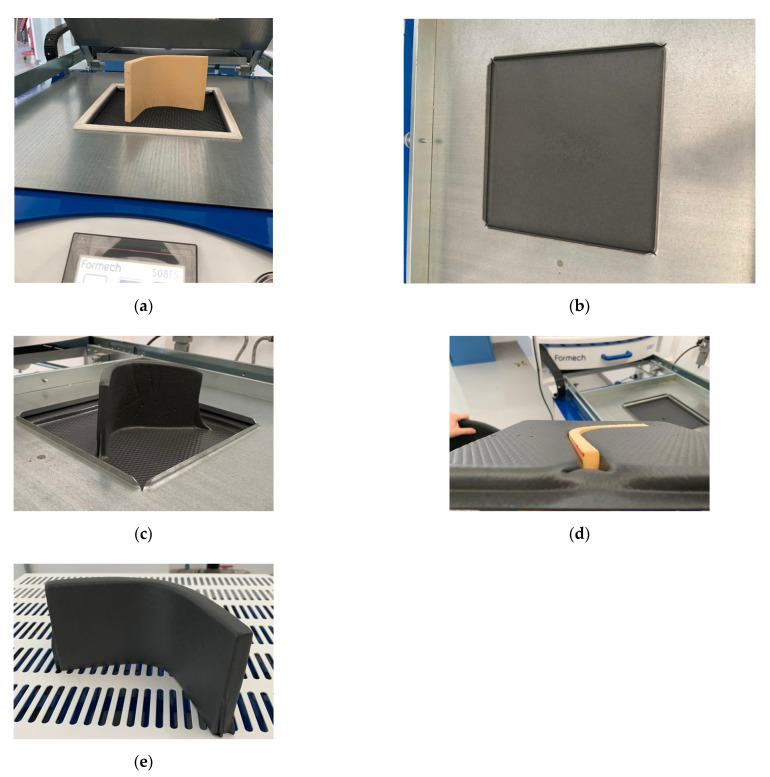
Schematic representation of the V-shaped vacuum forming panels: (**a**) foam core lays on the work platform, (**b**) ABS sheet places in the reduced processing window, (**c**) ABS-T/Foam/ABS-T panel, (**d**) bottom of the vacuum forming panel, and (**e**) panel after removing the excess of material. The panel is covered with the ABS sheet.

The compressed molded ABS sheets with 30 × 30 × 1 mm^3^ were used for the thermoformed foam panels ABS-T/Foam/ABS-T. The foam was formed with the heat gun as described previously in Figure 4. The foam acts as a mold, and it was laid in the working platform base. The ABS sheets were placed in the reduced processing window of the Formech 508FS, and the system was closed. The quartz heaters transfer the heat to the ABS sheet, and the auto-level display avoids material sag during the heating cycle and maintains an even distance between heater and sheet. When the ABS sheet is ready to form, the working platform base releases, the vacuum starts, and the cooling fan is activated. This procedure is summarized in Figure 13.

Vacuum forming is an alternative manufacturing process to the development of aircraft cabin interior panels. The technology is faster and cheaper than compression molding. Furthermore, the cores act as molds and are covered for all sides, which can be helpful for one-step decorative processes and avoid the use of tapestries. However, the vacuum process does not offer self-adhesion between the mold and the plastic sheets, and the ABS is not entirely compatible with polyurethane foam. Therefore, 3M™ Hi-Tack 71 spray adhesive was applied to the foam and ABS sheets before the vacuum process (ABS-TA/Foam/ABS-TA).

**Figure 14:**
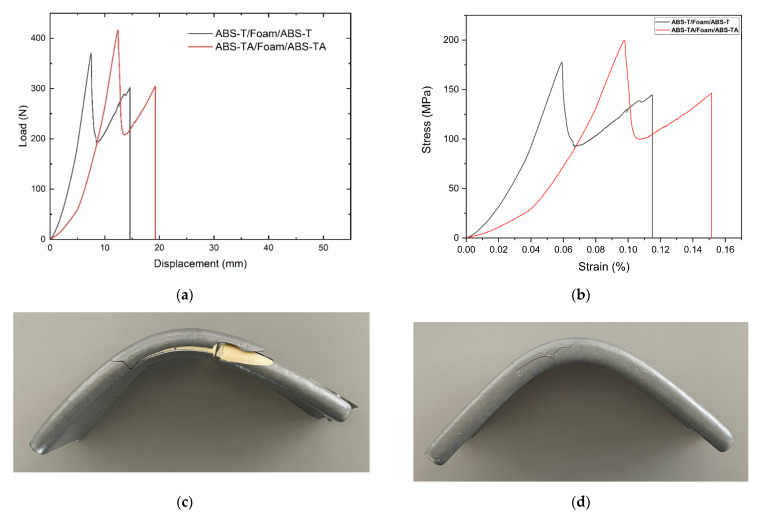
Mechanical behavior and failure: (**a**) representative load vs. displacement curves, (**b**) representative stress vs. strain curves registered during the bending tests, (**c**) failure of the ABS-T/Foam/ABS-T panels, and (**d**) failure of ABS-TA/Foam/ABS-TA panels. The pictures present the damages that occurred during the test.

The mechanical properties of ABS-T/Foam/ABS-T and ABS-TA/Foam/ABS-TA panels were evaluated according to the ASTM C393, with a span of 120 mm due to the restrictions in the ABS sheets size, and a crosshead speed of 1 mm/min. Figure 14 shows the mechanical response, and the mechanical parameters are presented in Table 2.

It can be observed that there are no significant variations in the shape of the curves, which allows us to suggest that both panels have similar mechanical behavior. However, according to the mechanical parameters obtained from the mechanical tests (Table 2), the standard deviation of the normal strength results considerably higher in the ABS-TA/Foam/ABS-TA than the ABS-T/Foam/ABS-T, which should imply that the adhesive could be influencing the mechanical parameters in a non-homogeneous way. In any case, the spray adhesive interferes with the mechanical behavior of the panels manufactured with the vacuum forming process as the ABS remains adhere to the foam, which is an interesting result as, assuming an impact on the panel during service, the aircraft interior panel maintains its aesthetics until it is removed or repaired (Figure 14d).

## 5. Conclusions

In previous work (Innovation in aircraft cabin interior panels part I: Technical assessment on replacing the honeycomb with structural foams and evaluation of optimal curing of prepreg fiberglass, under review), the use of analytical tools such as DSC and DMA allowed a complete evaluation of the curing conditions of fiberglass prepreg in the manufacture of sandwich composites. That knowledge allows us to evaluate the feasibility of replacing the honeycomb and fiberglass skin layers core with rigid polyurethane foams and thermoplastic polymers.

In this work, the sandwich composites were transformed into complex V-shaped panels. The structural composites formed with HC and fiberglass showed higher mechanical properties than the proposed sandwich composites, although the new composites fit with the non-structural applications.

The vacuum forming process proposed and analyzed in this work was feasible for developing non-structural panels. This manufacturing technique is fast, easy, economical, and ecological as it uses recyclable materials. The vacuum forming also covers the entire panel and avoids subsequent rework such as upholstery and painting of the panels. The results lead to more detailed research on materials and manufacturing processes that may be feasible in developing and optimizing panels for aircraft cabin interiors.

## Figures and Tables

**Table 1:** Description of the panel configurations evaluated in this work.

Panel Configuration	Faces	Core	Representation
GF/HC/GF	Glass fiber prepreg	Honeycomb	Reference panels
GF/Foam/GF	Glass fiber prepreg	Foam	Experimental panels
ABS/Foam/ABS	Thermoplastic ABS	Foam	Disruptive panels

**Table 2:** Bending parameters.

Composite Panel	Max Normal Strength (MPa)	Max Shear Strength (kPa)	Max Deflection (mm)
GF/HC/GF	17.44 ± 0.62	57.5 ± 0.62	13.80 ± 0.62
GF/Foam/GF	2.22 ± 0.62	56.1 ± 0.62	41.39 ± 0.62
GF/gridding foam/GF	0.90 ± 0.62	62.3 ± 0.62	37.87 ± 0.62
ABS/HC/ABS	6.52 ± 0.02	240.3 ± 0.12	32.71 ± 2.50
ABS/Foam/ABS	10.48 ± 0.03	390.4 ± 0.10	34.29 ± 0.43
ABS/gridding foam/ABS	5.14 ± 0.87	170.2 ± 0.09	51.65 ± 2.63
ABS-T/Foam/ABS-T	9.29 ± 1.47	320.2 ± 5.96	15.87 ± 3.71
ABS-TA/Foam/ABS-TA	7.13 ± 2.68	253.6 ± 8.53	15.37 ± 2.31

## Data Availability

This study did not report any data.
